# Supplementary material for: Effects of Catfish Egg Lectin on Cancer Cells Differ According to the Globotriaosylceramide Species They Express
Source: Int J Mol Sci. 2025 Sep 23;26(19):9278. doi: 10.3390/ijms26199278 (PMC12525471; doi:10.3390/ijms26199278)
Supplement: Supplementary file 1 [file ijms-26-09278-s001.zip › ijms-3806624-supplementary.pdf]

Supplementary Data

Effects of Catfish Egg Lectin on Cancer Cells Differ According to the Globotriaosylceramide Species They Express

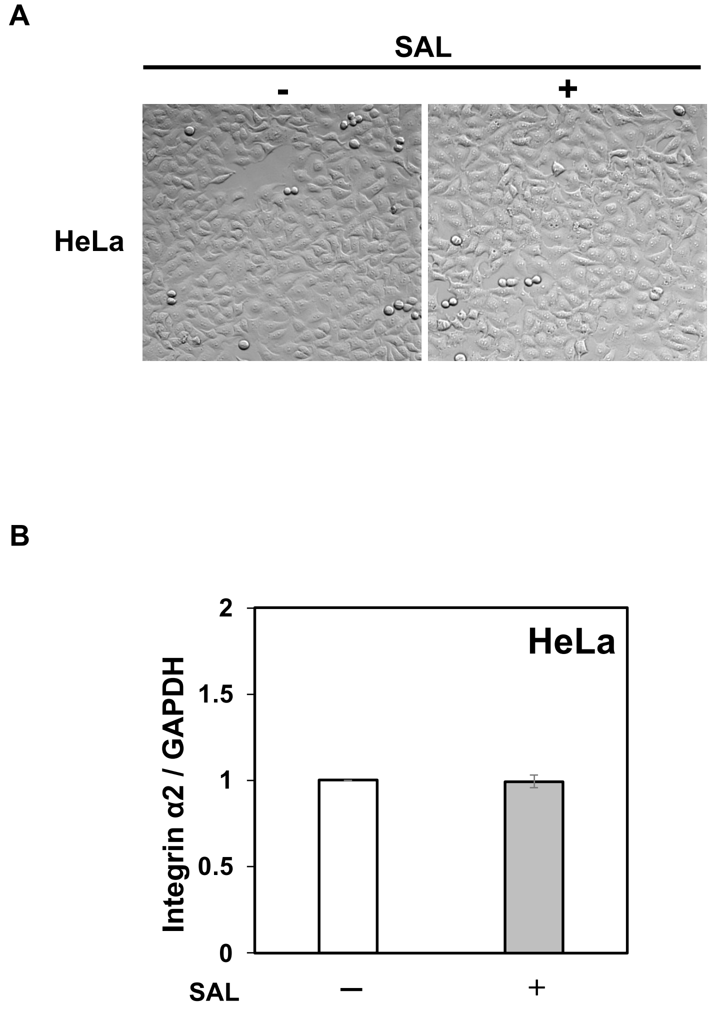

Supplementary Figure S1. ***Silurus asotus*** **(Amur catfish) egg lectin (SAL) treatment does not exert morphological changes or alter integrin α2 expression in HeLa cells.** (A) Morphological changes in HeLa cells induced by treatment with SAL (50 µg/mL) for 48 h at 37 °C. Negative control [SAL (-)]: addition of phosphate-buffered saline (PBS) instead of lectin. The images are from bright-field microscopy captured using a 10× objective lens. (B) Total RNA extracted from HeLa cells (5×10^4) was analyzed using reverse transcription quantitative PCR with specific primers for integrin α2 and *GAPDH*. The control value was defined as 1. Fold increases in target genes relative to the control were normalized to the level of *GAPDH*.

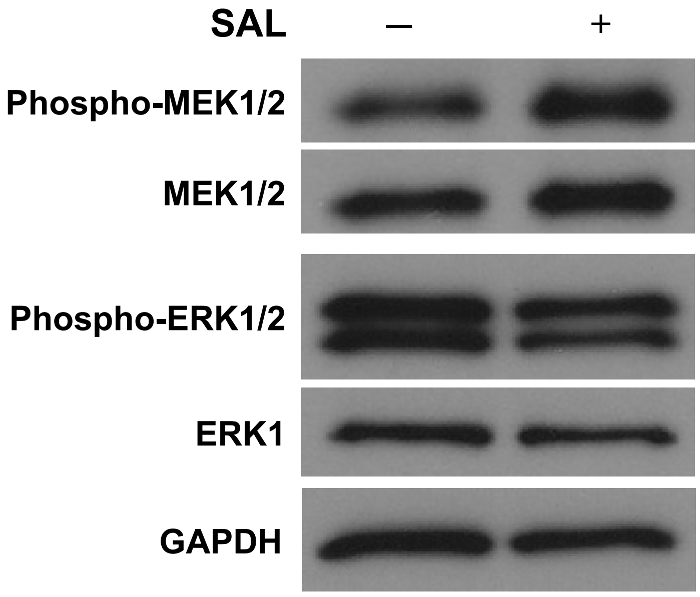

Supplementary Figure S2. SAL treatment does not induce the phosphorylation of mitogen-activated protein kinase kinase (MEK) and extracellular signal-regulated kinase (ERK). JKT-1 cells (1×10^6) were treated with SAL (50 µg/mL) at 37 °C for 48 h. Whole-cell extracts were subjected to western blotting using antibodies against phospho-MEK1/2, MEK1/2, phospho-ERK1/2, ERK1, and GAPDH. Representative results from triplicate experiments are shown.

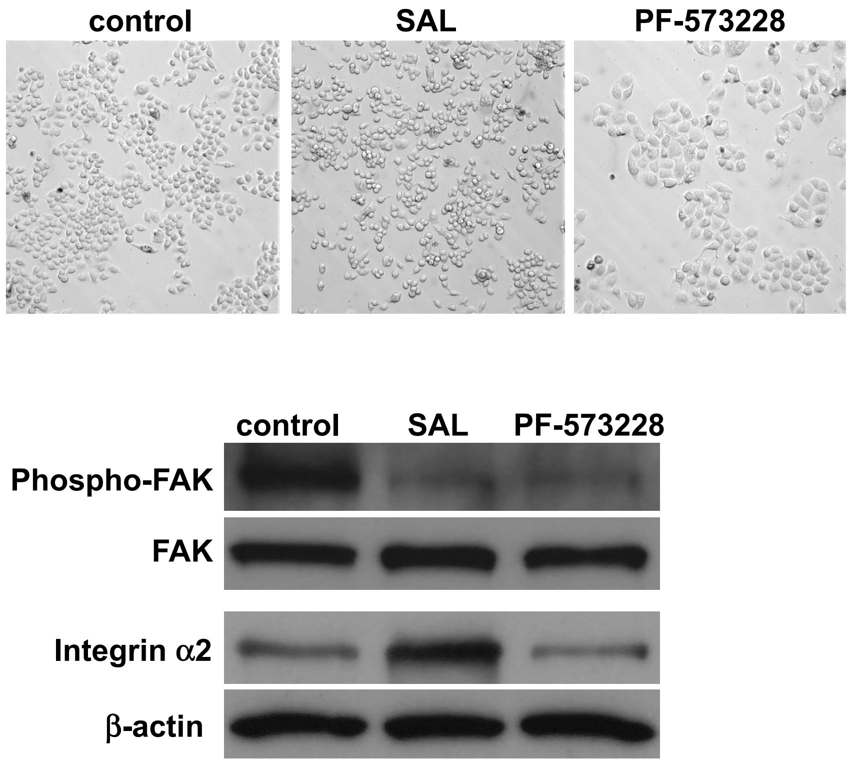

Supplementary Figure S3. Morphological changes and integrin α2 expression in SAL- or PF-573228-treated JKT-1 cells. (A) Morphological changes in JKT-1 cells induced by treatment with SAL (50 μg/mL) for 48 h at 37 °C or PF-573228 (10 μM) for 2 h at 37 °C. For negative control, PBS was added instead of SAL or PF-573228. The images are from bright-field microscopy captured using a 10× objective lens. (B) Degree of expression and phosphorylation of FAK and integrin α2 proteins as detected by western blotting. Aliquots (10 μg) of whole cell lysates were loaded onto sodium dodecyl sulfate-polyacrylamide gel electrophoresis SDS-PAGE gels and transferred to a polyvinylidene difluoride (PVDF) membrane. Phospho-FAK, FAK, integrin α2, and β-actin were detected by chemical luminescence following treatment with the respective primary antibodies and horseradish peroxidase (HRP)-conjugated secondary antibodies, as described in the Materials and Methods of the main text.

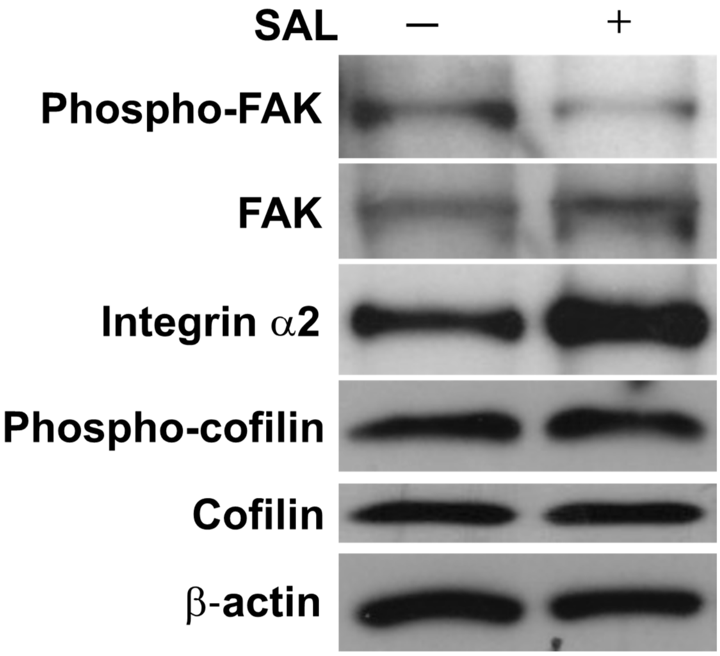

Supplementary Figure S4. SAL treatment does not induce the phosphorylation of cofilin. JKT-1 cells (1×10^6) were treated with SAL (50 μg/mL) at 37 °C for 48 h. Whole-cell extracts were subjected to western blotting using antibodies directed against phospho-FAK, FAK, integrin-α2, phosphor-cofilin, cofilin, and β-actin. Representative results from triplicate experiments are shown.
